# Silver Nanoparticle Conjugation-Enhanced Antibacterial Efficacy of Clinically Approved Drugs Cephradine and Vildagliptin

**DOI:** 10.3390/antibiotics7040100

**Published:** 2018-11-15

**Authors:** Abdulkader Masri, Ayaz Anwar, Dania Ahmed, Ruqaiyyah Bano Siddiqui, Muhammad Raza Shah, Naveed Ahmed Khan

**Affiliations:** 1 Department of Biological Sciences, School of Science and Technology, Sunway University, Bandar Sunway 47500, Malaysia; abdul@gmail.com or 17025941@imail.sunway.edu.my (A.M.); ayazanwarkk@yahoo.com (A.A.); ruqaiyyahs@sunway.edu.my (R.B.S.); 2 HEJ Research Institute of Chemistry, International Center for Chemical and Biological Sciences, University of Karachi, Karachi 74600, Pakistan; dania.ahmed48@yahoo.com (D.A.); raza_shahm@yahoo.com (M.R.S.)

**Keywords:** Cephradine, Vildagliptin, nanoparticles, antibacterial, nanotechnology

## Abstract

This paper sets out to determine whether silver nanoparticles conjugation enhance the antibacterial efficacy of clinically approved drugs. Silver conjugated Cephradine and Vildagliptin were synthesized and thoroughly characterized by ultraviolet visible spectrophotometry (UV-vis), Fourier transform infrared (FT-IR) spectroscopic methods, atomic force microscopy (AFM), and dynamic light scattering (DLS) analysis. Using antibacterial assays, the effects of drugs alone and drugs-conjugated with silver nanoparticles were tested against a variety of Gram-negative and Gram-positive bacteria including neuropathogenic *Escherichia coli* K1, *Pseudomonas aeruginosa*, *Klebsiella pneumoniae*, methicillin-resistant *Staphylococcus aureus* (MRSA), *Bacillus cereus* and *Streptococcus pyogenes*. Cytopathogenicity assays were performed to determine whether pretreatment of bacteria with drugs inhibit bacterial-mediated host cell cytotoxicity. The UV-vis spectra of both silver-drug nanoconjugates showed a characteristic surface plasmon resonance band in the range of 400–450 nm. AFM further confirmed the morphology of nanoparticles and revealed the formation of spherical nanoparticles with size distribution of 30–80 nm. FT-IR analysis demonstrated the involvement of Hydroxyl groups in both drugs in the stabilization of silver nanoparticles. Antibacterial assays showed that silver nanoparticle conjugation enhanced antibacterial potential of both Cephradine and Vildagliptin compared to the drugs alone. Pretreatment of bacteria with drugs inhibited *E. coli* K1-mediated host cell cytotoxicity. In summary, conjugation with silver nanoparticle enhanced antibacterial effects of clinically approved Cephradine. These findings suggest that modifying and/or repurposing clinically approved drugs using nanotechnology is a feasible approach in our search for effective antibacterial molecules.

## 1. Introduction

Infectious diseases are a significant burden on public health, driven largely by socio-economic, environmental and ecological factors [1]. About 15 million of 57 million annual deaths worldwide are estimated to be caused by infectious diseases, principally due to bacterial pathogens [1]. The burden of morbidity and mortality falls most heavily on people in developing countries [2]. Drug-resistant microbes are a major factor causing microbial re-emergence [3]. Among numerous bacteria, *Escherichia coli*, *Staphylococcus aureus*, *Pseudomonas aeruginosa*, *Bacillus* species, *Klebsiella pneumoniae* are important human pathogens contributing to urinary tract infections, neonatal meningitis, gastroenteritis, wound and skin diseases, food poisoning and nosocomial infections [4,5,6,7,8,9,10]. The overuse of antibiotics has contributed to bacterial acquisition of drug resistance resulting in reduced efficacy of available drugs.

During the past decade, nanomedicine has shown great potential due to effectiveness of various nanoconjugates against pathogenic microbes [11]. Nanomaterials have been frequently used as effective coatings to prevent bacterial adhesion to surfaces as well as bactericidal agents [12]. He et al., showed the development of self-defensive and antibacterial adhesion surface coating based on bilayer hydrogel which can promote cell adhesion and proliferation [13,14]. Polymers-based antibacterial agents are also an important class of nanomaterials. Yuan et al. reported various types of hydroxyl-rich cationic derivatives of star-like poly (glycidyl methacrylate) as broad-spectrum antibacterial and antifouling surface coating agents [15]. Antibacterial activity of low molecular weight cationic polymers is shown to affect the membrane permeability and disruption against a broad range of bacteria [16]. In another report, a salivary statherin protein inspired poly(amidoamine) dendrimer is shown to exhibit antibacterial effects as effective coating on hydroxyapatite [17,18]. Similarly, dendrons have been shown as a clicking tool for generating nonleaching antibacterial materials [19]. Metal nanoparticles have been studied extensively because of their unique physicochemical characteristics including catalytic activity, optical properties, electronic properties, antimicrobial activity, and magnetic properties [20]. Among these, silver nanoparticles (AgNPs) have shown growth inhibitory as well as bactericidal effects [21]. The high surface area of AgNPs leads to high antimicrobial activity as compared with the silver metal [22]. With the limited discovery of novel antibacterial agents, a feasible approach is to modify clinically approved drugs to enhance their efficacy and/or drug repurposing to expedite discovery of effective formulation of antibacterial agents.

Cephradine (relative molecular mass 349.406 g mol^−1^) is a first generation cephalosporins antibiotic drug that is widely used in the treatment of bacterial infections of the urinary and the respiratory tract, as well as ear, skin and soft tissues. It is used against both Gram-positive and Gram-negative bacteria. Its mode of action is inhibition of bacterial cell wall synthesis [23,24]. Vildagliptin (relative molecular mass 303.399 g mol^−1^) is an antidiabetic drug, which is a small molecule and inhibits dipeptidyl peptidase-4 (DPP4). Vildagliptin has been shown to stimulate insulin secretion and inhibit glucagon secretion in a glucose-dependent manner [25,26,27]. Here we tested whether conjugation of AgNPs can enhance efficacy of the clinically approved drug, Cephradine.

## 2. Materials and Methods

### 2.1. Bacterial Cultures

The cultures of six bacterial isolates including neuropathogenic *Escherichia coli* K1 (a cerebrospinal fluid isolate from a meningitis patient; 018:K1:H7), strain E44, was used in the present study (Malaysian Type Culture Collection 710859), and methicillin-resistant *Staphylococcus aureus* (MRSA) was used as described previously (Malaysian Type Culture Collection 381123). The MRSA strain was originally derived from the blood cultures, obtained from the Luton & Dunstable Hospital NHS Foundation Trust, Luton, England, UK. *Pseudomonas aeruginosa*, *Klebsiella pneumoniae*, *Bacillus cereus* and *Streptococcus pyogenes* were obtained from the Microbiology Research Laboratory at Sunway University. Stock cultures were refreshed by subculturing every 15 days on nutrient agar plates and were maintained at 4 °C.

### 2.2. Synthesis of AgNPs Coated with Drugs

Cephradine conjugated silver nanoparticles (Ceph-AgNPs) were synthesized. Briefly, 5 mL (0.1 mM) Cephradine aqueous solution was reacted with 5 mL (0.1 mM) silver nitrate aqueous solution, and the reaction mixture was magnetically stirred for 10 min. Twenty μL of 5 mM freshly prepared Sodium borohydride aqueous solution (NaBH_4_) was added in the above stirring reaction mixture. The color of solution turned yellow-brown from transparent upon addition of a reducing agent indicating the reduction of silver ions and the formation of Ceph-AgNPs [28,29]. For Vildagliptin-conjugated silver nanoparticles (Vgt-AgNPs), a similar procedure was repeated by optimizing different volume ratio (*v*/*v*) of silver solution and drugs. Stable Vgt-AgNPs were obtained at respective *v*/*v* of silver to drug at 1:1. The amount of drug loaded on the nanoparticles was also measured. Nanoparticles were centrifuged at 12,000× *g* for 1 h, supernatant was collected, freeze-dried, and the unloaded drugs was determined by weighing. The results are expressed as the percentage of the drug amount contained in 100 mg of the dried nanoparticle. The percentage of drug loading on nanoparticles was found to be 52% and 68% for Ceph-AgNPs and Vgt-AgNPs, respectively.

### 2.3. Characterization of AgNPs-Coated Drugs

After successful synthesis of nanoconjugates, Ceph-AgNPs and Vgt-AgNPs were subjected to complete analysis via ultraviolet-visible spectrophotometry (UV-vis), Fourier transformation infrared (FT-IR), atomic force microscopy (AFM), and dynamic light scattering (DLS) as described previously [28,29].

### 2.4. Bactericidal Assay

Antibacterial potential of AgNPs, Ceph and Ceph-AgNPs was determined by using bactericidal assay [30]. Briefly, bacterial cultures were adjusted to optical density (OD) of 0.22 at 595 nm using a spectrophotometer (OD_595_ = 0.22) which corresponds to 10^8^ colony-forming units per mL (C.F.U. mL^−1^). An inoculum of 10 μL of above bacteria culture (equivalent to approximately 10^6^ C.F.U.) was incubated with various concentrations of either Ceph-AgNPs, and Vgt-AgNPs in 1.5 mL centrifuge tubes at 37 °C for 2 h. For negative controls untreated bacterial culture were incubated with phosphate buffer saline (PBS). Vildagliptin and Cephradine alone were used as additional controls, while bacteria incubated with 100 μg mL^−1^ of gentamicin were used as positive control. Next, bacteria were serially diluted and 10 µL of each dilution was plated on nutrient agar plates. These plates were incubated at 37 °C overnight, followed by viable bacterial C.F.U. count.

### 2.5. Cytopathogenicity Assay

Cytopathogenicity assays were performed as described previously [31]. Briefly, *E. coli* K1 were incubated with various concentrations of Cephradine, Vildagliptin, and their nanoconjugates for 2 h at 37 °C. Next, all test samples were incubated with confluent HeLa monolayers in supplemented medium. Plates were incubated at 37 °C for 24 h in a 5% CO_2_ incubator and observed for cytotoxic effects. At the end of this incubation period, the supernatants were collected and cytopathogenicity was detected by measuring lactate dehydrogenase (LDH) release (Cytotoxicity Detection kit) as follows: % cytotoxicity = (sample value − control value)/total LDH release − control value) × 100. Control values were obtained from host cells incubated in RPMI-1640 medium alone. Total LDH release was determined from HeLa cells treated with 1% Triton X-100 for 30 min at 37 °C. The basis of this assay is that cell supernatant containing LDH catalyzes the conversion of lactate to pyruvate, generating reduced form of nicotinamide adenine dinucleotide (NADH) and H^+^. In the second step, the catalyst (diaphorase, solution from kit) transfers H and H^+^ from NADH and H^+^ to the tetrazolium salt p-iodo-nitrotetrazolium violet (INT), which is reduced to formazan (dye), and absorbance is read at 490 nm.

## 3. Results

### 3.1. Characterization of Cephradine and Vildagliptin Coated Silver Nanoparticles

**Figure 1:**
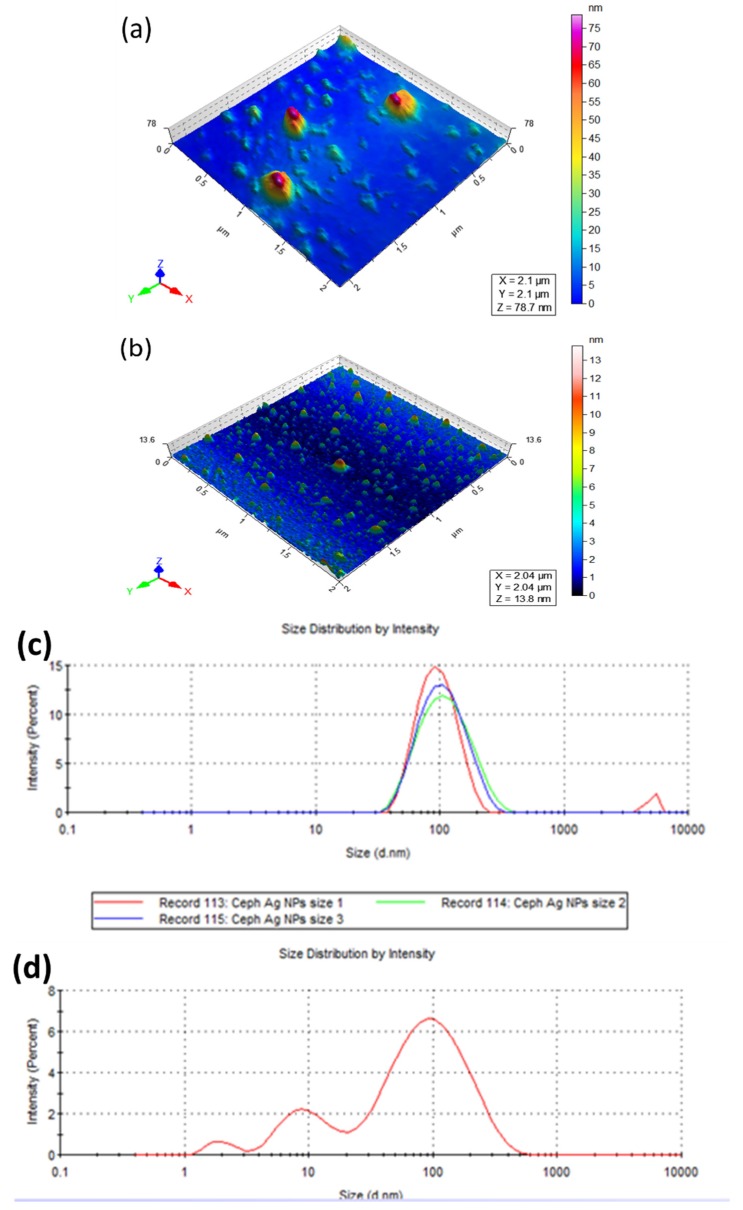
Representative images of atomic force microscopy (AFM) of Ceph-AgNPs (**a**); Vgt-AgNPs (**b**); AFM topographs were recorded on Agilent 5500 instrument used in tapping mode with silicon nitride cantilever. Ceph-AgNPs were found to be 85 nm (**c**); whereas, Vgt-AgNPs with the size distribution of 33 nm as measured by DLS (**d**). Both nanoparticles were spherical and polydisperesed.

**Figure 2:**
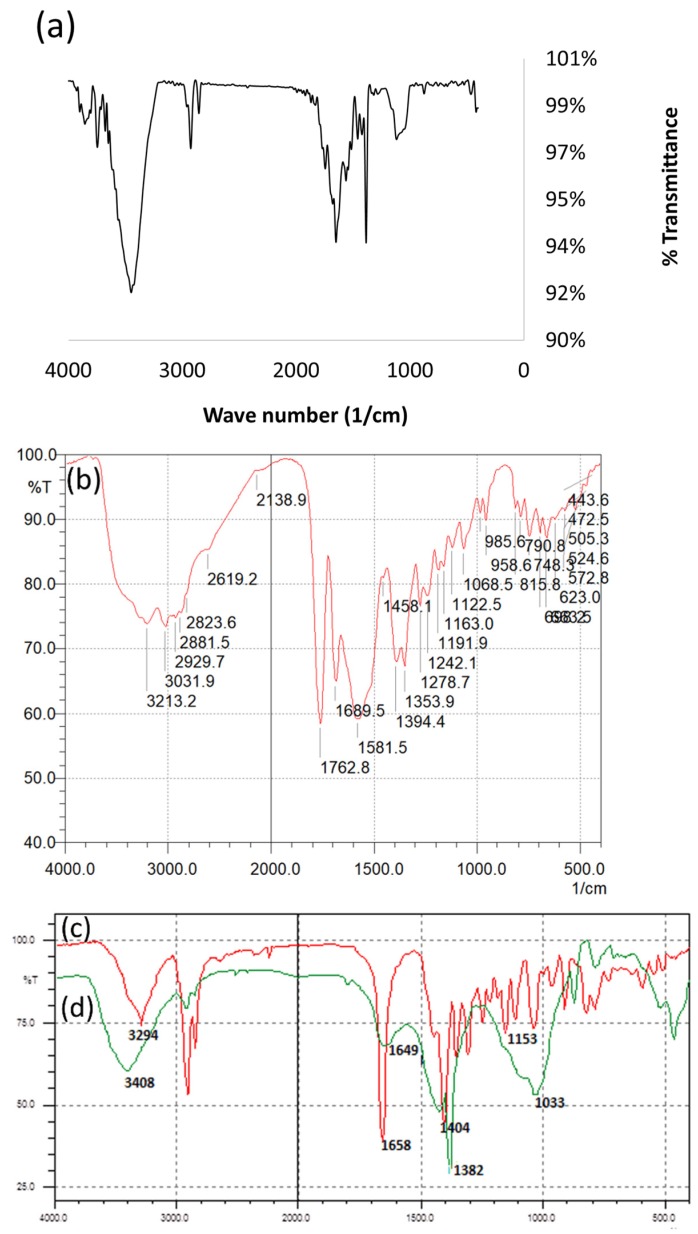
Representative spectra of FT-IR of Ceph-AgNPs (**a**); Vgt-AgNPs (**b**); shows the presence of hydroxyl, lactam, carboxylic acid and amino groups. However, while compared with drugs alone (**c**,**d**), hydroxyl groups peaks showed a shift in wave number which indicates the interaction with silver nanoparticles. Spectra were recorded at Bruker Vector 22 instrument using Potassium bromide (KBr) disc method.

The UV-vis spectra of both silver-drug nanoconjugates showed characteristic surface plasmon resonance band in the range of 400–450 nm. AFM images were recorded to ascertain the morphology of these nanoparticles. The representative topographical images of Ceph-AgNPs and Vgt-AgNPs revealed the formation of spherical nanoparticles (Figure 1a,b). The size distribution of drugs nanoconjugates was determined by DLS (Figure 1c,d). Vgt-AgNPs was found to be a mixture of small and large particle size ranging from 2, 7, and 90 nm with an average size of 33 nm, as compared to Ceph-AgNPs (average size 85 nm). FT-IR analysis demonstrated the involvement of hydroxyl groups in both drugs in the stabilization of silver nanoparticles (Figure 2).

### 3.2. Cephradine and Vildagliptine Conjugated with AgNPs Exhibited Increased Bactericidal Effects against E. coli K1 and MRSA Compared with AgNPs and Drugs Alone

**Figure 3:**
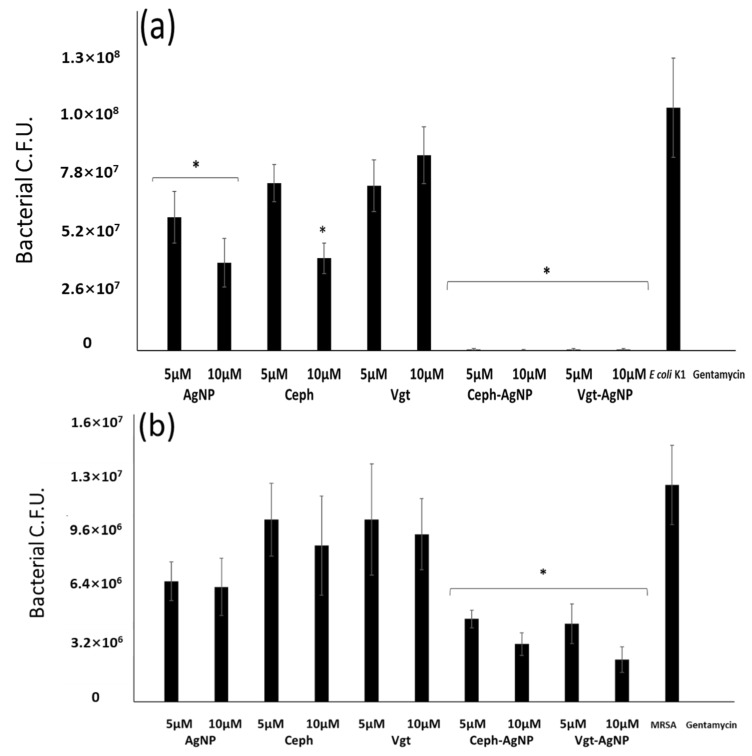
*E. coli* K1 (**a**); MRSA (**b**); colonies were determined following incubation with drugs alone, Ag alone, AgNPs conjugated with drugs. Briefly, 1 × 10^6^ *E. coli* K1, MRSA colonies were incubated with drugs and controls at 37 °C for 24 h. Next, colonies were accounted. Both drug-conjugated AgNPs exhibited significant bactericidal effects (*p* < 0.05 using *t*-test, two-tailed distribution, as indicated by asterisk). The results are the mean ± standard error of three independent experiments performed in duplicate.

Bactericidal assay was performed to determine the effects of drugs conjugated with AgNPs and drugs alone on *E. coli* K1 and MRSA. The concentrations of samples were adjusted to achieve MIC50 (minimum inhibitory concentration to kill 50% bacteria), which can be observed in MRSA and *S. pyogense*, and in the rest of bacteria MIC90 (minimum inhibitory concentration to kill 90% bacteria) is observed at tested concentrations. The results revealed that both drugs conjugated with AgNPs exhibited significant bactericidal effects (*p* < 0.05 using *t*-test, two-tailed distribution). In addition, treatment with bare AgNPs alone had limited effects on *E. coli* K1 and MRSA (Figure 3). Notably, Ceph-AgNPs showed significant bactericidal effects at 5 and 10 μM compared with drugs alone (*p* < 0.05). Vildagliptin alone did not show significant bactericidal effects at 5 or 10 μM, while, at 5 and 10 μM, Vgt-AgNPs, bacteria were significantly reduced (from 7.3 × 10^7^ to 5.4 × 10^5^) and (from 8.6 × 10^7^ to 4.8 × 10^5^) respectively for Gram-negative *E. coli* K1, and (from 1.0 × 10^7^ to 4.3 × 10^6^) (from 9.3 × 10^6^ to 2.3 × 10^6^) at 5 and 10 μM for Gram-positive MRSA (Figure 3).

### 3.3. Cephradine and Vildagliptine Conjugated with AgNPs Exhibited Increased Bactericidal Effects against P. aeruginosa, K. pneumoniae, B. cereus, S. pyogenes Compared with the Drugs Alone

**Figure 4:**
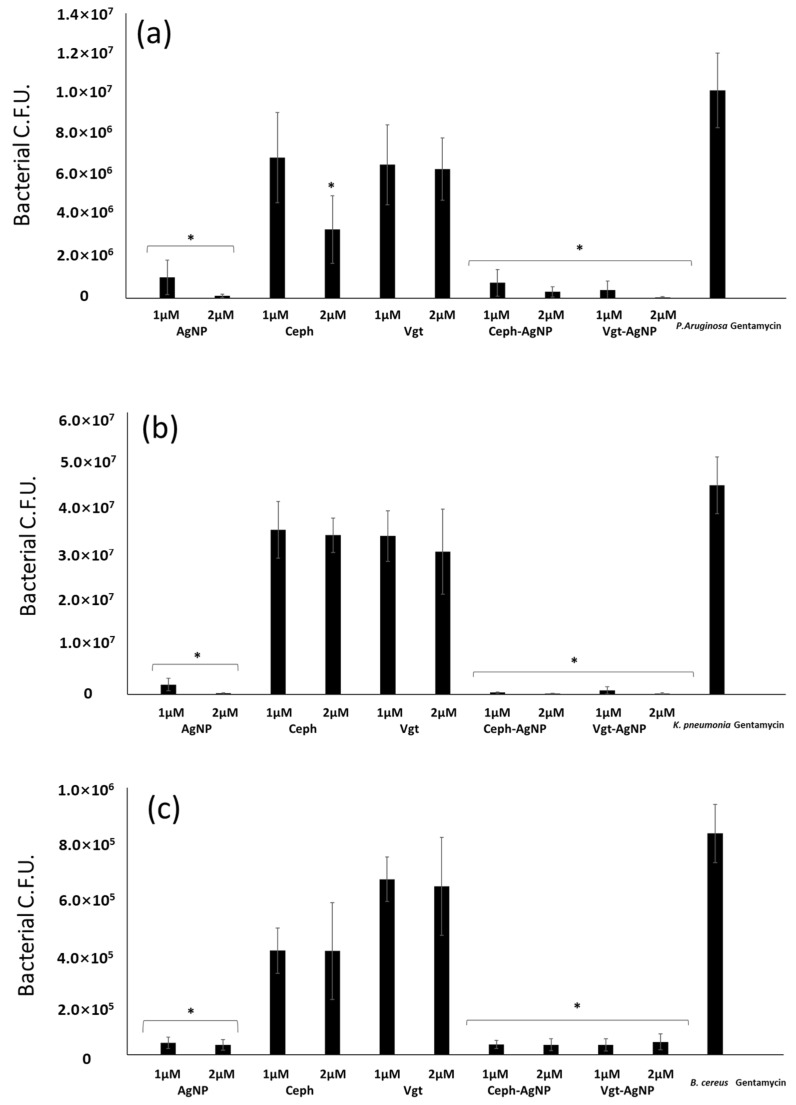

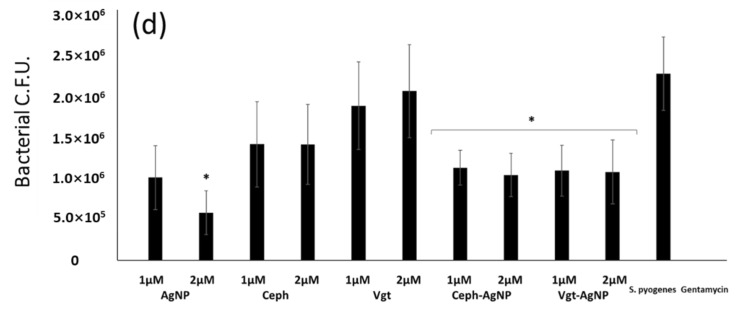
*P. aeruginosa* (**a**); *K. pneumoniae* (**b**); *B. cereus* (**c**); *S. pyogenes* (**d**). Bacterial colonies were determined following incubation with Cephradine and Vildagliptine. Briefly, 1 × 10^6^ bacteria were incubated with drugs and controls at 37 °C for 24 h. Both drugs—conjugated AgNPs and Ag-NPS—alone exhibited significant bactericidal effects (*p* < 0.05 using *t*-test, two-tailed distribution, as indicated by asterisk), while as there was no significant effects of drugs alone. The results are the mean ± standard error of three independent experiments performed in duplicate.

The results revealed that drugs conjugated with AgNPs exhibited significant bactericidal effects at 1- and 2-μM concentration against *P. aeruginosa*, *K. pneumoniae*, *B. cereus*, *S. pyogenes* (*p* < 0.05 using T test and two-tailed distribution). However, at 1 μM and 2 μM concentration, the treatment with AgNPs alone had similar effects on the number of bacteria. Notably, Ceph-AgNPs and Vgt-AgNPs showed significant effects at 1 μM and 2 μM compared with the drugs alone (Figure 4).

### 3.4. Silver Nanoparticle-Conjugated Drugs Inhibited E. coli K1-Mediated Host Cell Cytotoxicity

**Figure 5:**
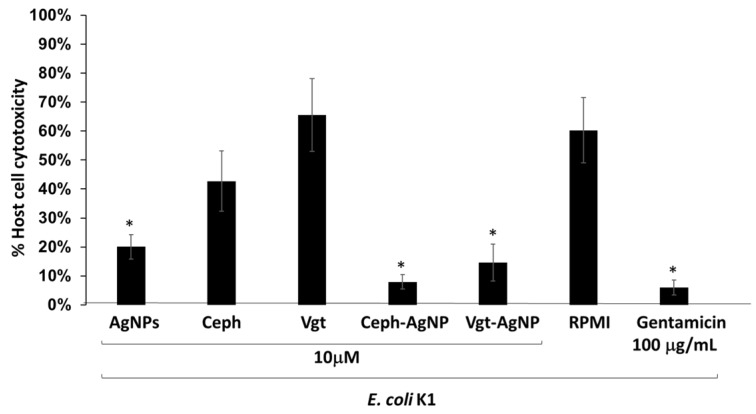
Cytopathogenicity assays were performed using *E. coli* K1 as described in materials and methods. Briefly, bacteria were incubated with test samples for 2 h before putting on HeLa cells monolayer with and without different drugs (10 µM), Cephradine (Ceph) and Vildagliptine (Vgt). Cells were then incubated for 24 h on standard conditions. Untreated bacteria, and bacteria treated with gentamicin were also treated with cells to evaluate their effects on cells cytotoxicity. Triton-X was used to lyse cells before determination of lactate dehydrogenase (LDH) release as marker of cell damage. LDH was determined by using Roche applied sciences LDH kit supplemented with enzyme and buffer. The % cytotoxicity was calculated by formula: % cytotoxicity = (sample value − control value)/total LDH release − control value) × 100. The results are representative of at least three independent experiments performed in duplicates. Asterisk indicates *p* < 0.05 using *t*-test, two-tailed distribution.

To determine whether AgNP-conjugated drugs inhibited bacteria-mediated host cell cytotoxicity, assays were performed by incubating 10^6^ *E. coli* K1 with HeLa cells for 24 h. *E. coli* K1 alone produced 60% host cell death. On the other hand, bacteria pretreated with AgNPs as well as Ceph-AgNPs and Vgt-AgNPs caused minimal host cell damage and host cell cytotoxicity was reduced to less than 15% (Figure 5). These findings also showed that Ceph-AgNPs and Vgt-AgNPs alone had minimal host cell cytotoxicity.

## 4. Discussion

The lack of development and approval of new and effective antibacterials as well as growing MDR microbes presents a major challenge in our ability to counter bacterial infections [31]. Nanotechnology offers great promise in the field of biomedicines, especially diagnosis and drug delivery. It offers opportunities for therapeutic agent delivery to specific cells and receptors. Nanomaterial-based drug delivery systems have the potential to improve pharmacokinetics and pharmacodynamics of the drugs [32]. The small size of nanoparticles provides them a greater surface area for maximum drug loading as well as high accessibility for specific targets. Recently, various drug-conjugated nanoparticles are being developed against infections caused by resistant microbes [33]. The most common metal carriers for nanoparticle-based drug delivery systems include gold, silver, and iron oxide due to their inertness and biocompatibility [33].

Though the mode of action of silver nanoparticles on the bacteria has been suggested to affect morphological and structural changes in the bacterial cells, the large surface area, provides better uptake by microorganisms [34]. Hence, silver nanoparticles have the ability to anchor to the bacterial cell wall and subsequently penetrate it, thereby causing structural changes leading to increased permeability of the cell membrane and cell death. In addition, the formation of free radicals by the silver nanoparticles have the ability to damage the cell membrane and make it porous resulting in bacterial cell death [35]. The bacterial membrane contains sulfur-containing proteins and the AgNPs interact with these proteins in the cell as well as with the phosphorus containing compounds. When AgNPs enter the bacterial cell, it forms a low molecular weight region in the center of the bacteria to which the bacteria conglomerates thus protecting the DNA from silver ions. Also, it generates reactive oxygen species, which are produced to attack the respiratory chain, cell division, and finally leading to cell death [36].

Silver conjugated Cephradine (mode of action involves binding and inactivation penicillin binding proteins leading to inhibition of peptidoglycan layer and causing cell lysis) and Vildagliptin (DPP4 inhibitor) were synthesized by reducing silver nitrate with sodium borohydride in the presence of drugs. These nanodrug conjugates were characterized by UV-visible spectrophotometry, FT-IR spectroscopy, and AFM. Cephradine and Vildagliptin successfully stabilized AgNPs and displayed surface plasmon resonance band in the range of 400–450 nm. FT-IR analysis showed the interaction of hydroxyl groups of drugs with silver nanoparticles for stabilization. Hence, the mode of stabilization is anticipated to be noncovalent interactions. Ceph-AgNPs and Vgt-AgNPs both were found to be spherical in shape and lie in a range of size distribution from 30–80 nm. Ceph-AgNPs were larger as compared to Vgt-AgNPs.

After characterization, these nanoparticles were subjected to antibacterial assays. Ceph-AgNPs and Vgt-AgNPs showed significant bactericidal effects against Gram-negative *E. coli* K1, *P. aeruginosa*, *K. pneumonia* and Gram-positive MRSA, *B. cereus*, and *S. pyogenes*. Nevertheless, these findings show tremendous potential in the development of new antibacterial formulations. As discussed previously, Cephradine is a cephalosporin first-generation antibiotic, which is not very effective against a number of bacteria because the different resistance mechanisms varied from the variation of the penicillin-binding protein, production of β-lactamase, existence of β-lactamase genes on plasmids or on bacterial chromosomes, and efflux pump mechanisms. For instance, MRSA produces abnormal penicillin binding protein 2A, which has low methicillin affinity (mediated through the *mecA* gene), which is carried on the staphylococcal cassette chromosome mec (SCCmec) by horizontal gene transfer and this results in resistance to Cephradine as an antibiotic [37]. Ceph-AgNPs provide a change in susceptibility of the drug along with, presumably, enhanced bioavailability. On the other hand, Vildagliptin (dipeptidyl peptidase-4 inhibitor) and its silver nanoparticles showed some promising effects as an antibacterial. Furthermore, these nanoparticles significantly and selectively reduced the pathogen-mediated host cell cytotoxicity caused by Gram-negative bacteria.

Drug-loaded nanoparticles enter in the bacteria by endocytosis showing either specific or non-specific type of interactions with cell membrane [38]. The positive charge of AgNPs interacts with lipopolysaccharides of Gram-negative bacteria with more affinity than the cellular wall of Gram-positive bacteria, that possess few sites for interactions, then releases the drugs intracellularly [38]. In previous reports, cephalosporin conjugated with AgNPs also showed enhancement in the antibacterial potency of Ceftriaxone and Cefixime against *E. coli* and *S. pyogenes*, respectively [39,40]. In conclusion, Cephradine and Vildagliptin showed bactericidal effects against the six tested bacteria in this study, but their conjugation with AgNPs enhanced their antibacterial efficacy. Moreover, Ceph-AgNPs and Vgt-AgNPs also significantly reduced the host cells cytotoxicity. The exact mechanism of action of these nanoparticles is not precisely understood and it is the subject of future studies along with testing their potential in vivo.
